# HIV Competition Dynamics over Sexual Networks: First Comer Advantage Conserves Founder Effects

**DOI:** 10.1371/journal.pcbi.1004093

**Published:** 2015-02-05

**Authors:** Bence Ferdinandy, Enys Mones, Tamás Vicsek, Viktor Müller

**Affiliations:** 1 Department of Biological Physics, Eötvös Loránd University, Budapest, Hungary; 2 MTA-ELTE Statistical and Biological Physics Research Group, Eötvös Loránd University and the Hungarian Academy of Sciences, Budapest, Hungary; 3 MTA-ELTE Theoretical Biology and Evolutionary Ecology Research Group, Eötvös Loránd University and the Hungarian Academy of Sciences, Budapest, Hungary; 4 Parmenides Center for the Conceptual Foundations of Science, Pullach/Munich, Germany; University of Bern, SWITZERLAND

## Abstract

Outside Africa, the global phylogeography of HIV is characterized by compartmentalized local epidemics that are typically dominated by a single subtype, which indicates strong founder effects. We hypothesized that the competition of viral strains at the epidemic level may involve an advantage of the resident strain that was the first to colonize a population. Such an effect would slow down the invasion of new strains, and thus also the diversification of the epidemic. We developed a stochastic modelling framework to simulate HIV epidemics over dynamic contact networks. We simulated epidemics in which the second strain was introduced into a population where the first strain had established a steady-state epidemic, and assessed whether, and on what time scale, the second strain was able to spread in the population. Simulations were parameterized based on empirical data; we tested scenarios with varying levels of overall prevalence. The spread of the second strain occurred on a much slower time scale compared with the initial expansion of the first strain. With strains of equal transmission efficiency, the second strain was unable to invade on a time scale relevant for the history of the HIV pandemic. To become dominant over a time scale of decades, the second strain needed considerable (>25%) advantage in transmission efficiency over the resident strain. The inhibition effect was weaker if the second strain was introduced while the first strain was still in its growth phase. We also tested how possible mechanisms of interference (inhibition of superinfection, depletion of highly connected hubs in the network, one-time acute peak of infectiousness) contribute to the inhibition effect. Our simulations confirmed a strong first comer advantage in the competition dynamics of HIV at the population level, which may explain the global phylogeography of the virus and may influence the future evolution of the pandemic.

## Author Summary

The African epicentre of the HIV pandemic is home to a vast array of divergent viruses; however, local epidemics in other parts of the world are typically dominated by a single variant (subtype) of the virus, with different subtypes found in the different regions. This pattern indicates that local epidemics outside Africa have been started by the introduction of single “founder” viruses in the susceptible populations. However, how these patterns persisted over several decades in the face of international migration requires further explanation. By analyzing simulated epidemics, we demonstrated that an epidemic established by the first successful founder strain can inhibit the introduction and slow down the subsequent spread of further virus strains by several mechanisms of interference. Our results have implications for the global evolution of the HIV pandemic: the fast expansion of subtypes benefited from a “first comer advantage,” and founder viruses may have been selected by random sampling, rather than due to superior transmissibility/fitness; the fast expansion of these (possibly) suboptimal virus strains may have considerably delayed the spread of more transmissible HIV variants; however, the future evolution of the pandemic is likely to be characterized by a slow expansion of viral strains with increased transmission potential.

## Introduction

The global pandemic of Human Immunodeficiency Virus (HIV) infections is being driven mainly by the group M lineage of HIV-1, which crossed the species barrier from chimpanzees to humans about 100 years ago [1,2]. By the time it started to spread beyond its epicentre in Central Africa, the virus had already accumulated considerable sequence diversity [2], and distinct divergent clades initiated a series of rapid expansions that gave rise to the subtypes of HIV-1 group M [3,4]. The global molecular diversity of the pandemic still bears the clear footprint of the strong founder effects that characterized this initial expansion. While diversity is very high near the epicentre of the epidemic in Central Africa, the epidemics of other regions are typically characterized by the dominance of at most a few subtypes or circulating recombinant forms (CRFs) [5]. The countries where more than one subtype is prevalent tend to be characterized by parallel, compartmentalized epidemics with distinct subtypes infecting different risk or ethnic groups [6–9], and transmission chains rarely cross national borders [10]. While the global spatial distribution of HIV subtypes is not completely static, the diversification of the epidemic and shifts between subtypes occur very slowly in most regions [5].

Understanding the factors that set the time scale of HIV competition dynamics at the population level has great practical relevance. Subtypes differ in both transmissibility [11–13] and the rate of disease progression [14,15], and further variation in these traits is bound to exist within the subtypes and in the vast diversity of unique recombinant forms (URFs) and unclassified basal lineages in Central Africa [16–18]. Virus variants that have higher transmission potential are likely to be spreading at the expense of less efficient strains, and epidemics may expand as the original variants are gradually replaced by “fitter” viral lineages. The risk and pace of these processes needs to be better characterized.

We developed a simple model of sexually transmitted HIV epidemics that allowed us to monitor the competition dynamics of distinct virus strains with varying rates of transmission. In sexually transmitted epidemics, HIV is transmitted over the network of sexual contacts, which tends to include a very limited subset of all possible contacts, i.e. the host population is very far from “free mixing”. To be able to detect effects arising from network structure, we implemented an individual based simulation that allowed us to track a dynamic network of contacts between the individuals, and to model HIV transmission along the links of the network. The model was parameterized based on data from generalized heterosexual epidemics in Africa.

## Results

Our model tracked a dynamic network of contacts between three types of nodes (individuals): male, female, and female sex worker (FSW). Stochastic processes were implemented for the formation and dissolution of links (heterosexual relationships); sexual acts over the links; transmission of HIV in serodiscordant acts; and death due to AIDS. Natural (non-AIDS related) turnover was implemented by tracking the age of individuals, and replacing individuals at the end of sexual activity; the network of contacts was parameterized based on contemporary data including variability in the promiscuity of individuals. The transmissibility of HIV depended on disease stage.

We simulated a simple scenario of competition between two strains of the virus. To assess the maximum potential for a “first comer advantage”, we started the simulations with one of the strains (the founder, or “resident” strain) and let the epidemics attain steady-state prevalence before introducing the second (“invader”) virus strain. The transmission rate of the invader strain was equal to or greater than that of the resident strain, and its chance and pace of growth was assessed in relation to its transmission advantage over the resident strain.

**Fig 1:**
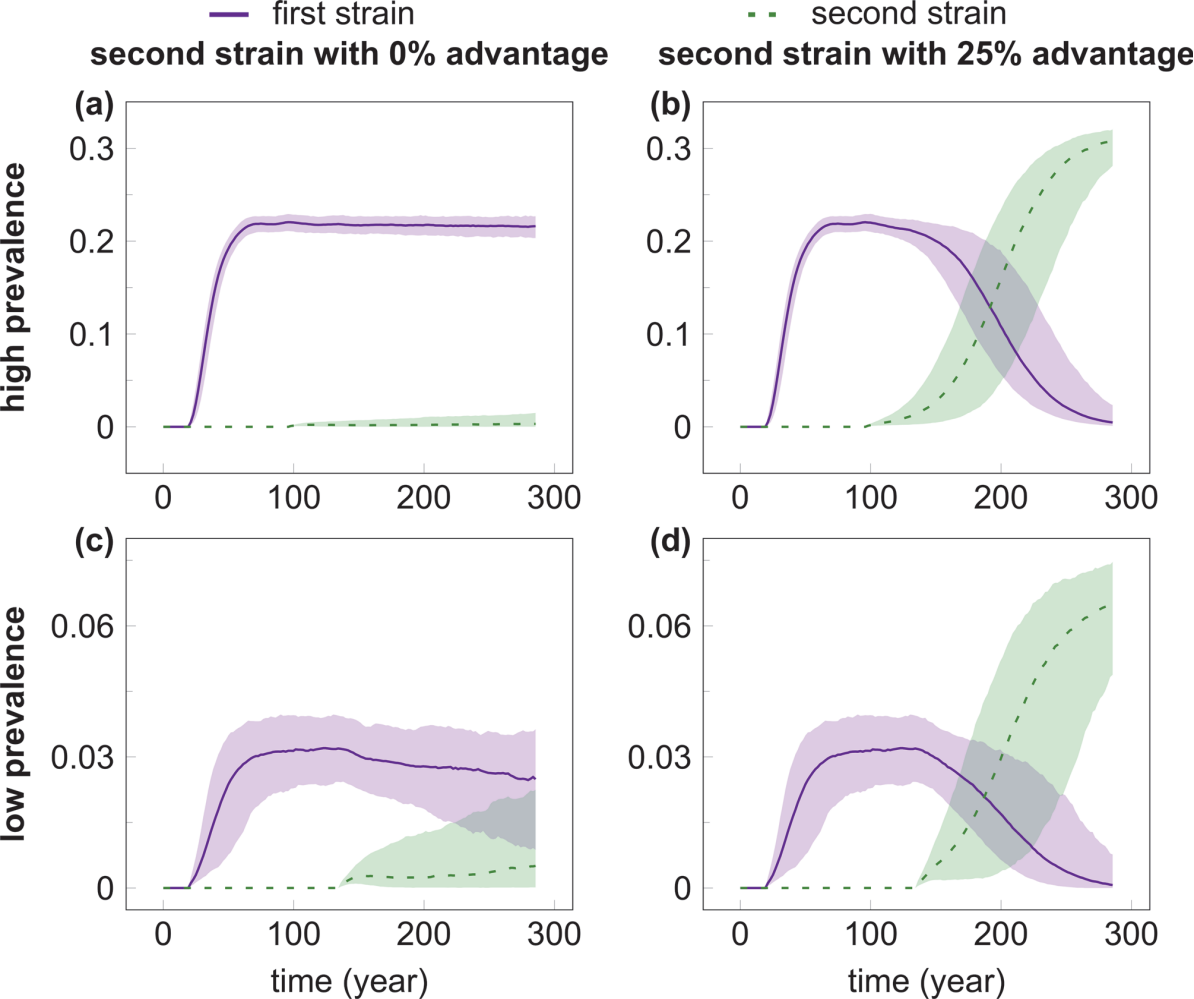
The time course of infection prevalence in multiple simulations of the competition of HIV strains. The invader virus had equal (A, C) or 25% greater (B, D) transmission rate compared with the resident strain in the high (A, B) or low (C, D) prevalence scenarios. The resident strain (solid purple line) was introduced in the population at Week 1000 (to allow the network to attain steady state); the invader strain (dashed green line) was introduced in the population when the first had already reached steady-state prevalence (at Week 5000 and 7000 for the high- and low-prevalence setting, respectively). Even with a 25% advantage in the transmission rate, it took the invader strain a median of 60 and 104 years to reach the prevalence of the resident strain in the low- and high-prevalence scenario, respectively. The lines show median prevalence from simulations where the invader strain did not go extinct (out of 1000 simulation runs); shading indicates the areas between the 5% and 95% quantiles. Simulation parameters were set as in Table 1.

We hypothesized that the effect on the spread of the invader strain may depend on the prevalence of the resident strain, and have therefore considered two scenarios, where the steady-state prevalence of the resident strain was around 0.03 and 0.2, respectively. The two scenarios were set by changing the baseline rate of transmissibility (see Materials and Methods/Parameters); all other parameters were kept constant. Fig. 1 shows the time course of multiple simulations for two selected cases where the invader virus had equal (Fig. 1A,C) or 25 percent greater (Fig. 1B,D) transmission rate compared with the resident strain in the high (Fig. 1A-B) or low (Fig. 1C-D) prevalence scenarios (S1 Fig. depicts cases with further values of the transmission rate advantage). The resident strain attains steady-state prevalence in about 84 and 74 years in the low- and high-prevalence scenario, respectively. With equal transmission rate, the invader strain shows no appreciable growth in a hundred years in the high-prevalence scenario (Fig. 1A), and grows, but remains in strong minority over the same time span in the majority of the simulations with the low-prevalence scenario (Fig. 1C). A 25 percent advantage in the transmission rate allowed the invader virus to outgrow the resident strain in both scenarios (Fig. 1B,D), but it still took a median of 60 and 104 years until the prevalence of the invader strain reached that of the resident strain in the low- and high-prevalence setting; due to its higher transmissibility, the invader strain was then able to attain higher steady-state prevalence compared with the initial steady state of the resident strain. Compared with the initial expansion of the resident strain, the expansion of the invader was much slower in all cases. In addition, 66.4 and 68.3 percent of the simulations with equal transmissibility of the invader resulted in the extinction of the invader virus in the low- and high-prevalence scenarios, respectively; extinction occurred in 2.4 and 1.2 percent of the cases when the invader had 25 percent transmission advantage. In contrast, with our settings the initial introduction of the resident virus was nearly always (in 998/1000 and 1000/1000 independent simulation runs of the low and high-prevalence settings, respectively) able to establish an epidemic that grew to steady state.

**Table 1:** Parameters used for the simulation of the model.

Symbol	Description	Value [reference]
N_m_	Number of men in the population	10000
N_f_	Number of women in the population	10000
γ_m_	Exponent of male degree distribution	2.45^a^ [71]
γ_f_	Exponent of female degree distribution	3.45^a^ [71]
K_c_	Number of clients per FSW per year	400^b^ [72–74]
κ_min_	Lower cutoff of annual degree distribution	1
κ_max_	Upper cutoff of annual degree distribution	1000^c^
p_b_	Probability of link breakup per week	0.05^d^ [73]
λ	Poisson parameter for the number of sex acts per week	2 [75,76]
ν_1_	Strain 1 per-contact transmissibility in chronic stage	0.001 or 0.002^e^ [77]
ν_2_	Strain 2 per-contact transmissibility in chronic stage	(1–1.5)*ν_1_
m_A_	Transmission multiplier for acute infection	9 [36]
T_acute_	Length of acute phase (weeks)	12 [20]
T_age_	Duration of sexual activity (years)	35 (age 15–50)
T_HIV_	Survival with HIV infection (range in years)	3–20 [78,79]
T_init_	Time steps (weeks) without virus	1000
T_single_	Time steps (weeks) with only one virus	6000 or 4000^e^

^a^Used to generate preferred annual contact degrees; for the exponents fitted to realized contact degrees, see Fig. 2 and S4 Fig.

^b^Middle value from 600 given in [72] and 150 calculated from [73,74].

^c^The maximum realized contact degree was lower in all simulations.

^d^Baseline rate for links between individuals with degree 1.

^e^Alternative values used to parameterize low- and high-prevalence settings.

### Mechanisms of interference

Our simulation framework allowed us to investigate three potential mechanisms of interference between the resident and the invader strains. First, infection with one HIV strain may afford some protection against superinfection with another strain: both the depletion of target cells and the induction of anti-HIV immune responses are likely to create less favourable conditions for infection compared with an uninfected individual. Because the strength of such an effect is still subject to debate (see Discussion), we used the following conservative approach in the default settings for our simulations. In a sexual act between two individuals who were both infected but with different strains of the virus, both strains had a chance to be transmitted in a two-step procedure. The first step tested successful initial transmission, which had a probability based on the transmission rate of the given strain, equivalent to the first infection of an uninfected individual. Then in the second step the superinfecting virus replaced the original strain with a probability based on the relative transmission rates of the two strains: in 50% of the cases if both strains had equal transmission rate, and with smaller or greater probability if the superinfecting strain had lower or higher transmission rate, respectively, compared with the original strain (see Materials and Methods for details). In this scenario, the first strain had neither advantage nor disadvantage at the within-host level, and the “inhibition effect” arose only from the assumption that the infection of each individual is dominated by a single virus strain, implying replacement rather than coexistence upon superinfection (which is a reasonable simplification for the modelling of population level spread; see Discussion). This algorithm reduced the average probability of superinfection to 50% of that of initial infection, which is consistent with a recent prospective cohort study that estimated about two-fold lower hazard of superinfection compared with initial infection [19]. However, we also tested a “dual infection” scenario, in which superinfection was completely unhindered, and both strains were able to co-exist within one individual after superinfection occurred. In this scenario, in sexual acts between a dual infected and an uninfected individual both virus strains had an independent probability of being transmitted.

Second, we hypothesized that the peak of infectivity that characterizes acute infection [20,21] may not occur again upon superinfection due to depleted target cell levels and the presence of anti-HIV immune responses. If this is indeed the case then the first virus strain to colonize a population may take advantage of a rapid early wave of expansion fuelled by a high relative frequency of efficient acute stage transmissions in a largely susceptible population (the relative contribution of acute stage transmissions in our simulations is shown in S2 Fig.). In contrast, any subsequent “invader” strain is limited to the lower rates of chronic transmission that characterize mature epidemics ([22] and S2 Fig.), and even successfully superinfected individuals represent a diminished resource if acute peak infectivity cannot be repeated. We implemented this possible mechanism by keeping track of disease stage independently of the identity of the infecting strain. If superinfection occurred after the end of acute infection, the individual was assumed to remain in chronic stage and the onward transmission of the superinfecting strain occurred according to its baseline (chronic) transmission rate. When superinfection occurred during the acute stage of the initial infection, then the superinfecting strain received the benefit of enhanced acute-stage transmission for the remaining time of the acute stage, timed from the initial infection of the individual. However, some evidence indicates that superinfection can generate a new temporary peak of viremia at least in some of the cases [23]. We have therefore also tested a scenario where superinfection started a new window of enhanced acute-stage infectivity.

**Fig 2:**
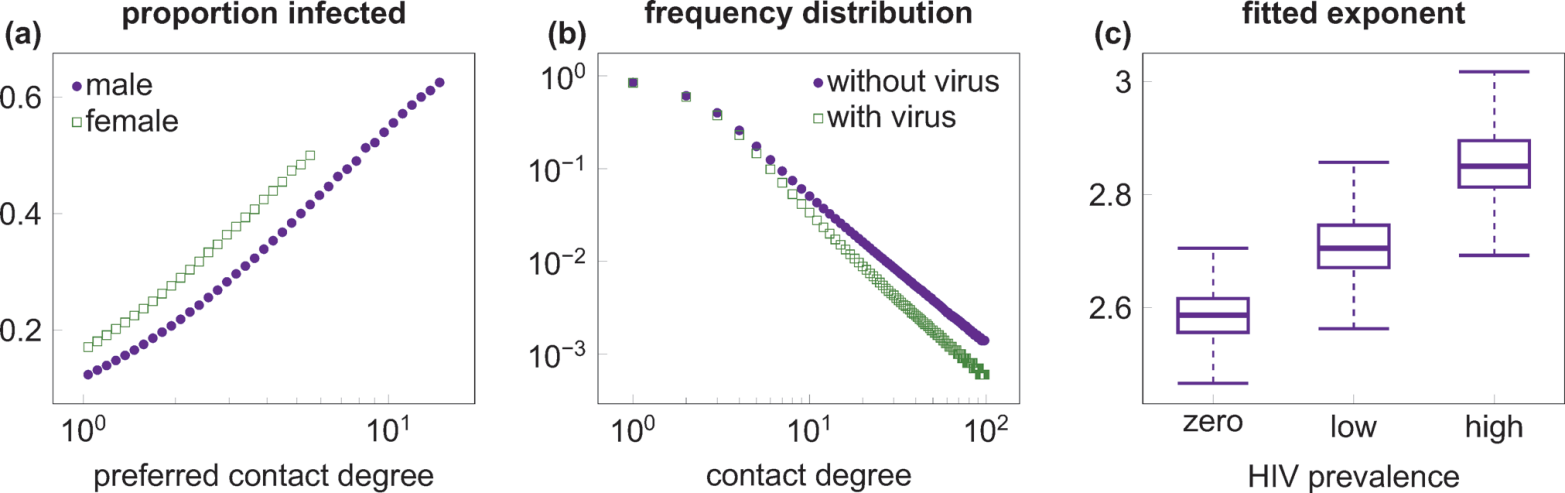
Preferential infection and killing of highly promiscuous individuals. (A) The ratio of infection among men (purple dots) and (non-FSW) women (green squares) increased with preferred contact degree (number of partners per year; plotted on logarithmic scale). The plot was created from 1000 independent simulation runs of single-strain epidemics of high prevalence, using logarithmic binning, right-censored at the top 1% of the male/female population (where rare classes result in strong stochastic variation). (B) The frequency distribution of the annual number of sexual contacts (realized contact degree) of males in uninfected populations (purple dots) and in populations with high-prevalence epidemics (green squares), based on median data from 1000 simulation runs. Highly promiscuous individuals were selectively depleted in the presence of the virus. (C) Boxplot of the exponents of power-law distributions fitted to male individuals in batches of 1000 independent runs with no virus, low and high prevalence epidemics, respectively. Boxes depict interquartile range, median is indicated by horizontal lines within the boxes, and whiskers extend to the farthest values that are not more than 1.5 times the box width away from the box. Medians (and IQR) of the exponents were 2.59 (2.56–2.62), 2.70 (2.67–2.75) and 2.85 (2.81–2.90) in the absence of the virus and with low or high prevalence epidemics, respectively; all pairwise comparisons between the three scenarios were statistically significant (p<10^–10^; Wilcoxon rank sum test). Selective depletion among females is shown in S4 Fig. Simulation parameters were set as in Table 1.

Third, we hypothesized that, in the absence of broadly available antiretroviral treatment (ART), the first HIV epidemics may also have an impact by selectively infecting and killing highly promiscuous individuals who form the “hubs” of the network. Such individuals have been shown to be particularly important for the spread of sexually transmitted diseases [24], and they are likely to be infected preferentially due to their larger number of contacts. Indeed, in our simulations the probability of infection was strongly related to the promiscuity (preferred contact degree) of the individuals (Fig. 2A). Using collated data from 100 simulation runs, logistic regression against log transformed contact degree (controlling also for age and gender) estimated an effect size of 2.48 (95% CI: 2.46–2.50; p<10^-10^), implying that the odds of being infected increased by a factor of exp(2.48), i.e., about 12-fold for every order of magnitude increase in the preferred contact degree (see S3 Fig. for the model fit); the effect was robust also in regressions on individual simulation runs (effect size range in 100 simulations: 2.26–2.66; p<10^-10^ for all simulation runs). As a result, an established epidemic of the resident virus strain depleted highly connected nodes of the network preferentially: the power-law exponent of the contact degree distributions (fitted to the actual number of partners in the last year) increased significantly compared with the pre-epidemic steady state (p<10^–10^, Wilcoxon rank sum test; Fig. 2B-C and S4 Fig.), which may also have put any invader strain at a disadvantage. To assess the strength of this effect, we also implemented a scenario in which all individuals who died of AIDS were replaced by an uninfected individual with the same promiscuity (preferred contact degree) as that of the deceased individual, which preserved the degree distribution of the contact network irrespective of the epidemics.

Our strategy was thus to construct a default simulation scenario using settings that we deemed most plausible (partially inhibited superinfection, with strain replacement when superinfection is successful; one-time acute peak of infectiousness; and emergent preferential depletion of highly connected individuals), then test the effect of switching off one mechanism at a time in a series of test scenarios: i) “dual infection” with possible co-existence of the two strains in the same individual and no inhibition of superinfection; ii) “multiple acute” with repeated episodes of enhanced acute-stage infectiousness upon each successful superinfection; and iii) “fixed degrees” in which the degree distribution of the contact network was preserved. This strategy allowed us to assess the relative impact of each mechanism on the population level competition dynamics, and served also as a sensitivity analysis for relaxing the assumptions of inhibited superinfection and one-time acute peak infectiousness.

### Inhibition of superinfection dominates first comer advantage

**Fig 3:**
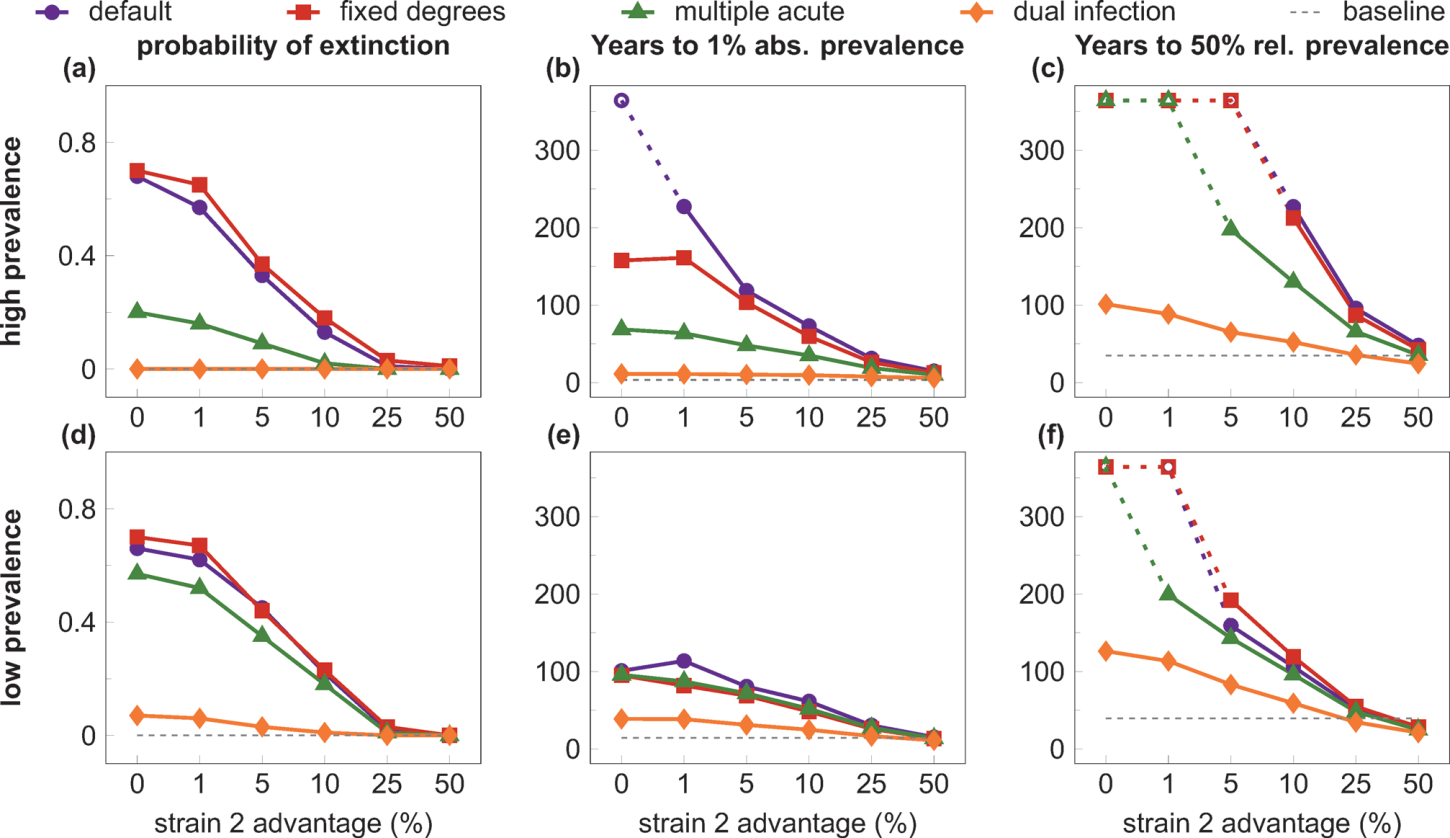
Quantifiers of the “first comer advantage”. All quantifiers are plotted against the relative transmission rate advantage of the second (invader) strain, with alternative scenarios to test interference mechanisms. Rows show results from the high (top row) and low prevalence (bottom row) settings; columns depict three different quantifiers; scenarios are coded by symbols and colour. In the default scenario (purple lines and dots) the invader strain faced a high risk of extinction (A, D) and experienced very slow growth to 1% absolute prevalence (B, E) and to 50% relative prevalence (C, F) at low values of transmission rate advantage, compared with the initial growth of the resident virus (dashed gray lines). The effect was largely abrogated with unhindered superinfection and co-existence (dual infection scenario; orange lines and diamonds), and, in the high-prevalence setting, partially mitigated by allowing for repeated “acute stage” peak infectivity after superinfection (multiple acute scenario; green lines and triangles); fixing the degree distribution of the contact network (fixed degrees scenario; red lines and squares) had little effect compared with the default scenario. Increasing the relative transmission rate advantage of the invader strain also decreased the inhibition effects: values comparable to the single-strain baseline were observed around 25%-50% transmission advantage. Data in B-C and E-F depict medians from 1000 simulation runs (excluding those where the invader virus went extinct). Parameters are listed in Table 1; scenarios are described in detail in the main text. The maximum length of simulations was 19,000 weeks (~365 years); empty symbols indicate where the invader strain did not reach the threshold prevalence by the end of the simulation in the majority of the cases.

We tested eight scenarios (default and three test cases, each in both low and high prevalence settings) with levels of relative transmission advantage for the invader strain ranging between 0–50 percent. The invader strain was introduced in the population when the resident virus had attained steady-state prevalence; all combinations of scenario and transmission advantage were tested in 1000 simulation runs. We extracted several statistics to quantify the probability and rate of the expansion of the invader virus (Fig. 3).

When the transmission advantage of the invader strain was small, most simulations of the default scenario resulted in the extinction of the invader variant in both the high (Fig. 3A) and the low (Fig. 3D) prevalence settings. In contrast, the first (resident) strain was able to establish a stable epidemic in nearly all (>99%; dashed gray line) simulation runs when introduced into a fully susceptible population, which indicates a strong first comer advantage at the early stages of the spread of new strains. Preserving the degree distribution of the contact network (“fixed degrees”) had negligible effect compared with the default scenario; allowing multiple peaks of acute-stage infectiousness substantially reduced the probability of extinction in the high, but not in the low-prevalence setting. Finally, allowing for unhindered superinfection and coexistence (“dual infection”) reduced the probability of extinction to near zero even with no transmission advantage, illustrating that the inhibition of superinfection was the major factor in the heightened extinction risk of the invader strain. Greater relative advantage in the transmission rate reduced the risk of extinction in all scenarios, approaching zero extinction risk at around 25 percent advantage.

We defined two more quantifiers based on the time it took the invader strain to grow to selected threshold levels (in both cases we derived the statistics from the simulation runs where the invader strain did not go extinct). The time to one percent absolute prevalence (infecting one percent of the total population) was selected as a low threshold that would allow for the detection of a new strain in a population (Fig. 3B,E). As a baseline comparison, we plotted also the median time until the resident strain attained one percent prevalence during its initial expansion (median of 14.4 and 3.3 years for the low and the high prevalence case; dashed gray lines). At small values of the transmission advantage, growing even to one percent prevalence can take a century or more in the default scenario (e.g. a median of 114 and 228 years with a transmission advantage of one percent, in the low and high-prevalence setting, respectively). The inhibition effect was stronger in the high-prevalence setting, and was gradually lost when the transmission advantage of the invader strain was increased to about 50 percent. The dominant mechanism of inhibition was again the inhibition of superinfection: allowing for dual infection abrogated most of the effect even at low values of the transmission advantage. The other two mechanisms of interference had negligible effect in the low-prevalence scenario (Fig. 3E), but had some partial effect in the high-prevalence scenario (Fig. 3B); multiple peaks of acute infectiousness had a stronger impact than fixed contact degrees also in this test case.

Finally, we also collected statistics on the time until the turning point when the invader strain accounted for 50 percent of the infections in the population (Fig. 3C,F). This time was extremely long (>300 years) when the invader strain had low transmission advantage in the default scenario, and a transmission advantage of 50 percent was needed to bring it down to a median of 27 and 48 years in the low and high-prevalence setting, respectively (in comparison, the resident strain reached 50% of its steady-state prevalence in a median of 35 and 40 years in the low and high-prevalence cases; dashed gray lines). Allowing for dual infection again had the strongest impact at lower transmission advantage, followed by allowing for multiple peaks of acute-stage infectiousness.

**Fig 4:**
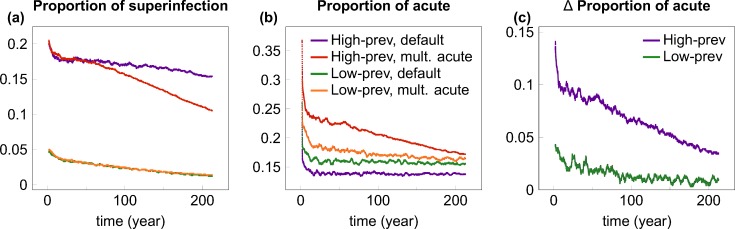
The contribution of superinfection events and acute-stage transmissions to the spread of the invader strain. (A) depicts the time course of the proportion of transmissions of the invader strain that involved superinfection of carriers of the resident virus. Coloured lines show smoothed proportion data for low and high prevalence epidemics using the default scenario, and the “multiple acute” scenario that allowed for repeated peaks of acute-stage infectiousness upon superinfection. In both scenarios, the contribution of superinfection was very low in the low-prevalence setting (green and orange lines), where most individuals were uninfected at the introduction of the invader strain; in contrast, many more transmissions involved superinfection in the high-prevalence setting (purple and red lines). (B) depicts the time course of the proportion of transmissions of the invader strain that originated from acute-stage transmitters in the four cases (colour coding is the same in A and B). (C) shows the difference in the proportion of acute-stage transmissions between the default and the multiple acute scenario for both prevalence settings (i.e. the distance between the red and purple, and between the green and yellow lines of Panel B). Allowing for multiple acute peaks of infectiousness greatly increased the proportion of acute-stage transmissions in the high-prevalence setting (purple line), but to a much lesser extent in the low-prevalence setting (green line). In all cases, time courses are plotted from the introduction of the invader strain into steady-state epidemics of the resident strain. Proportion data were calculated by combining transmission events recorded in 1000 simulation runs, then smoothed by averaging with a sliding window of length 100 weeks. Parameters were set as in Table 1; the transmission advantage of the invader strain was 5% in all cases.

To understand why the impact of repeated acute-stage infectivity depended on the initial prevalence of the resident strain, we calculated the contribution of superinfection events and acute-stage transmissions to the spread of the invader strain in the various scenarios (Fig. 4). As expected, the contribution of superinfection was very low (<5%) in the low-prevalence setting, where most individuals were uninfected at the introduction of the invader strain; in contrast, many more transmissions (~20% initially) involved superinfection of carriers of the resident virus in the high-prevalence setting (Fig. 4A). Because multiple acute peaks of infectiousness take effect only when superinfection occurs, their impact on the frequency of acute transmissions was much stronger in the high-prevalence setting (Fig. 4B-C), and the increased frequency of efficient acute transmissions explains the reduced risk of extinction and faster growth of the invader strain when multiple acute peaks of infectivity were allowed in the high-prevalence scenario. In the high-prevalence setting (Fig. 4A), the relative contribution of superinfection decreases faster in the multiple acute scenario compared with the default scenario: the reason for this difference is that multiple acute peaks of infectiousness can substantially accelerate the outgrowth of the invader strain in the high-prevalence scenario (S5 Fig.), and the decline of the resident strain results in a decreasing probability that the invader (super)infects an individual who carries the resident strain.

### Short head start or fast population turnover reduce first comer advantage

**Fig 5:**
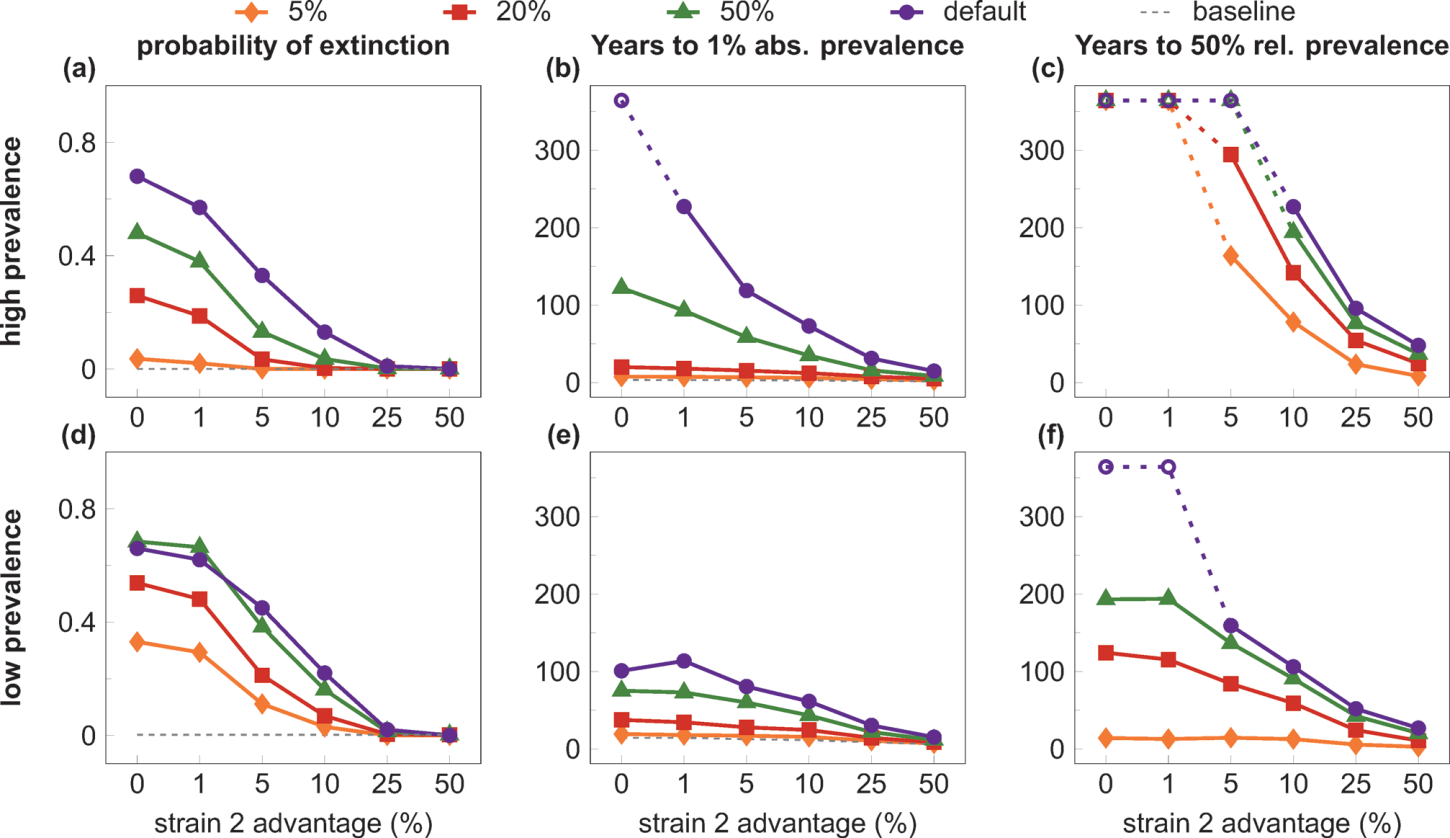
Quantifiers of the “first comer advantage” when the invader virus enters in the growth phase of the resident strain. Plotted are cases (coded by symbols and colour) where the invader was introduced when the resident strain had attained 5%, 20% or 50% of its plateau prevalence; in the default case the second virus was introduced at Week 7000/5000 in the low/high prevalence setting (as in Fig. 3) when the resident strain had already reached a stable plateau in its prevalence. All quantifiers are plotted against the relative transmission rate advantage of the second (invader) strain. Rows show results from the high (top row) and low prevalence (bottom row) settings; columns depict three different quantifiers. First comer advantage is weaker when the invader enters at earlier stages of the growth of the initial strain. Dashed gray lines in A-B and D-E represent the growth of the resident virus without competition; with early introduction of the invader strain, 50% relative prevalence in C and F is attained well below plateau prevalence and therefore cannot be compared to the 50% point of single-virus epidemics as a baseline. Data in B-C and E-F depict medians from 1000 simulation runs (excluding those where the invader virus went extinct). Parameters are listed in Table 1; competition dynamics followed the default scenario in all cases. The maximum length of simulations was 19,000 weeks (~365 years); empty symbols indicate where the invader strain did not reach the threshold prevalence by the end of the simulation in the majority of the cases.

We next investigated what happens if the invader strain enters the population when the first strain is still in its growth phase and has not yet reached steady-state prevalence. We ran simulations where the invader was introduced when the resident strain had attained 5%, 20% or 50% of its plateau prevalence level and compared the outcome to the previous default setting (Fig. 5). As expected the first comer advantage was weaker when the second strain was introduced early in the growth phase of the first strain. However, the probability of extinction of the invader strain increased substantially already when the resident strain was at only 5% of its plateau level initially in the low-prevalence setting (Fig. 5D), or at 20% of plateau level in the high-prevalence setting (Fig. 5A). The time to 50% relative prevalence of the invader strain was strongly affected when the resident strain was initially at 5% of its plateau level in the high-prevalence setting (Fig. 5C), and at 20% of plateau level in the low-prevalence setting (Fig. 5F). We thus conclude that (depending on the prevalence setting) some aspects of the first comer advantage are established relatively early in the initial expansion of the first successful strain.

We also tested the effect of faster population turnover using a residence time of 20 years (as opposed to the default of 35 years) for uninfected individuals in the population. This scenario may apply to regions that experience intense population movements and/or high rates of non-AIDS mortality. Faster population turnover had little effect on the initial risk of extinction for the invader strain, but could substantially accelerate the rate of its growth in the simulation runs where it did not go extinct (S6 Fig.). The probability of extinction is influenced by the instantaneous availability of susceptible individuals, which is not affected by the rate of turnover (at a fixed population size); however, subsequent growth depends on the continuous supply of new susceptibles, which increases with the rate of population turnover.

### Case study: The expansion of HIV-1 subtype A in Uganda

While the mechanisms of interference can slow down the invasion of new strains, the global pandemic is not static and major shifts between HIV lineages have been occurring in selected regions. The best-characterized example is the expansion of HIV-1 subtype A at the expense of subtype D in Eastern Africa [5,25,26], and we used the detailed data from Uganda [26] to derive a crude estimate for the transmission advantage required for the observed expansion. Between 1994 and 2002, the estimated prevalence of subtype D decreased from 11.9% to 8.1%, and the prevalence of subtype A increased from 2.8% to 3.0% in Uganda; the overall prevalence of HIV declined from 17% to 13% over the same period [26]. The overall decline probably reflects changes in risk behaviour and/or health interventions; with stable (17%) prevalence, the relative expansion of subtype A would roughly correspond to growing to 3.9% (~3*17/13) absolute prevalence, over a background prevalence comparable to that of our high-prevalence setting. Analyzing data from our high-prevalence default scenario, we found the closest match with the data when the transmission advantage of the invader strain was set to 25% (Fig. 1B), in which case the increase from 2.8% to 3.9% prevalence took 7.8 years on average (vs. 8 years in the empirical dataset). The rate of the relative expansion of subtype A observed in Uganda would thus require about 25% advantage over the resident subtype D strain, in a setting of stable overall prevalence in our simulations. Decreasing overall prevalence in the empirical data indicates a slowing turnover of infections, which requires a greater transmission advantage for the same tempo of strain replacement. This is roughly consistent with the independent empirical estimation that the overall (unadjusted) transmission rate of subtype A was 47% higher than that of subtype D in the same cohort [12].

## Discussion

Both the probability and the rate of epidemic growth were strongly reduced for virus strains introduced into the steady state of a resident epidemic, when the default assumptions of partially inhibited superinfection and one-time acute peak of transmissibility were used in our simulations. To outgrow the resident strain over a few decades (the time scale of human observations), the invader virus needed 25 percent or greater advantage in its rate of transmissibility over the resident strain. Of the three potential mechanisms of interference investigated, the direct inhibition of superinfection had the strongest effect in both prevalence settings, while one-time acute peak transmissibility had substantial effect only in the high-prevalence setting. The depletion of highly connected nodes in the network had little effect in most of the cases. In principle, a fourth mechanism of interference could also arise, because superinfected individuals (having progressed with their first infection) tend to have a shorter remaining lifespan, and therefore a shorter window of opportunity to transmit the superinfecting strain, compared with individuals who are infected for the first time. However, restarting the clock of disease progression upon superinfection had very little effect compared with the default scenario in a set of simulations (not shown); therefore, this mechanism does not seem to play an important role.

The reduction in the rate of growth of the invader strain was greater when the resident virus had higher initial prevalence, while the rate of extinction was insensitive to initial prevalence. The inhibition effect was weaker, but still considerable when the second strain was introduced while the first strain was still in the early phases of its growth, or if the (non-AIDS related) turnover of the population was faster.

Our results suggest that HIV competition dynamics is indeed characterized by a strong “first comer advantage” if the first strain to colonize a local transmission group expands to near plateau prevalence before further viral strains invade. This effect slows down the diversification of the epidemics and facilitates the persistence of founder effects. As far as we are aware, this is the first attempt to generally characterize the competition dynamics of different HIV strains over sexual networks, including multiple possible mechanisms of interference. The specific case of competition between HIV-1 and HIV-2 has been modelled in a similar framework [27], while another study looked at the competition of multiple evolving virus strains at the epidemic level without considering network structure [28]. Finally, Gross et al. [29] demonstrated that the inhibition of superinfection can preserve founder effects in the competition of equally transmissible virus strains, but have not considered network structure, alternative mechanisms of interference, or differential transmission.

The impact of the distinct mechanisms of interference may be modulated by factors that were not included in our simple model. First, heterogeneity may exist in the transmission rates across individuals and over time within the same partnership: in particular, per-contact transmission risk may decrease from the first exposures to subsequent contacts within a serodiscordant partnership (independent of the effect of acute infection) [30,31]. Such an effect can arise if the individuals highly susceptible to the virus of their partners tend to be rapidly infected, and the couples that remain serodiscordant become enriched in cases of low transmissibility over time (as reviewed in [32]). Similar effects are expected also if the partners of infected individuals can develop partially protective immunity to HIV in the exposures that do not result in transmission [33–35]. Irrespective of the mechanism, if time dependent variation in transmissibility is strain specific, then the invader virus has the advantage of being engaged in “first contact” with higher probability than the resident strain, which would decrease the first comer advantage of the latter. Second, if superinfection can generate a new “acute” temporary peak in viremia (and transmissibility) at least in some of the cases [23], then this mechanism of interference may also be weaker, which could reduce the first comer advantage (particularly in high-prevalence epidemics, according to our results). On the other hand, a detailed analysis of transmission risk in serodiscordant couples in Africa [20], and a recent phylodynamic analysis of a North American epidemic [21] have both estimated about 20-fold higher transmissibility during acute compared with chronic HIV infection. Using a 9-fold consensus estimate [36] we may thus have underestimated the interference effect if repeated “acute” peaks of transmissibility do not (or only rarely) occur after superinfection. Third, the observed partial inhibition of superinfection may not take effect until several months from the first infection [19,37], e.g., if partially protective immune responses and/or a limiting depletion of target cells take a longer time to develop [19]. This would allow unhindered superinfection in the first few months after seroconversion, which would reduce the first comer advantage, especially if the second strain arrives while the first epidemic is still in its growth phase. Fourth, there is considerable debate on the strength of the (partial) protection from superinfection. Several studies have found zero or very low rates of superinfection [38,39] (which would implicate strong protection against superinfection), while at the other extreme some studies have found rates of superinfection comparable to those of initial infection [37,40,41] (which would indicate little or no protection). The differences in the estimates may reflect genuine variation between the study populations, but also differences in study design, inclusion criteria and sensitivity of detection [23]. Importantly, deep sequencing methods allow the detection of superinfecting strains that grow only to low levels in the superinfected individuals (e.g., [40]), and may often be lost after a transient episode of superinfection [42]. Such low-level superinfection is likely to result in onward transmission of the minority variant with much lower probability compared with the baseline rate of transmission. In the context of population level spread and competition, superinfection is likely to be relevant only when the superinfecting strain grows to dominate the virus pool of the individual, which we approximated by allowing only strain replacement (and no co-existence) in the default scenario of our simulations. Relaxing this assumption and allowing for unhindered superinfection abrogated most of the first comer advantage in our results: we therefore conclude that the strong founder effects observed in the global phylogeography of HIV are more parsimoniously explained if superinfection is partially inhibited and the transmission of more than one strain from the same individual is rare.

Our generic modelling framework could not aim to account for all the (often population specific) complexities of human population dynamics and behaviour. For simplicity, population size was kept constant in our simulations, including instantaneous replacement of individuals who died of AIDS. With this implementation population turnover increased with HIV prevalence, e.g., the rate of death/replacement was about a third higher in the high-prevalence steady state compared with an uninfected population. Given that faster turnover reduces the first comer advantage, our results can be regarded as a conservative (under) estimation of the inhibition effect. Not replacing individuals who die of AIDS results in decreasing population size, which may further inhibit the expansion of invader strains by reducing the supply of susceptible individuals. In contrast, fast population growth or immigration may dilute the inhibition effect by increasing the influx of susceptible individuals. Migration may also play a role by introducing the same invading HIV strain repeatedly from a source population: this would eventually overcome the barrier of initial extinction, but would likely have little impact on the subsequent growth of the invader strain. Furthermore, an established HIV epidemic may also affect sexual behaviour: if high-risk sexual practices and/or promiscuity decrease in response to an ongoing epidemic, the spread of subsequent invader strains may be further inhibited. Finally, the complexities of the sexual network, e.g., assortative mating may further influence the strength of the inhibition effects.

Alternative or additional mechanisms may also contribute to the preservation of founder effects. If viral adaptation occurs to host traits that vary between human populations, then a locally adapted virus strain will enjoy a selection advantage against strains adapted to other host populations (as has been observed in some model systems of host-parasite interactions [43,44]). For example, the distribution of Human Leukocyte Antigen (HLA) alleles may differ between human populations, and local transmission may fix escape mutations against the locally frequent alleles that initially had a protective effect [45], particularly in populations with lower HLA diversity [46]; although this seems to be occurring slowly and to a limited extent where HLA diversity is high [47–49]. Location or population specific differences may exist in other host traits affecting HIV acquisition or transmission (e.g., in restriction factors [50,51] or in other components of innate immunity [52]). Each locally adapted virus strain may therefore have a competitive advantage within its established host population, and a disadvantage in other populations—which would also slow down the global mixing of variants or could even result in the long term survival of several virus strains in different populations. We note, however, that long-term co-existence of several virus strains in the same epidemic (connected transmission group) is possible only if specific conditions are fulfilled, e.g., strain-specific immunity or therapy creates frequency-dependent selection that favours the rare type. Without such specific conditions, the strain with the highest transmission potential in a given host population drives all other strains extinct in the long run: this principle of competitive exclusion holds true from simple abstract mathematical models [53,54] to complex simulations, including ours.

We parameterized our model based on data from generalized heterosexual epidemics in Africa, but it could easily be adapted to other routes of transmission and to concentrated epidemics. Furthermore, the results of our simulations can be applied not only to the competition of two distinct lineages (e.g., subtypes, or distinct clades of the same subtype [55]), but also to competition between virus variants that arise by local mutations. The general take-home message of our work posits that the expansion of the HIV pandemic to all susceptible populations across the world has made the conditions far less favourable for the spread of “novel” virus strains, irrespective of their origin.

Our results have important implications for understanding the past and for predicting the future of the HIV pandemic. The observed first comer advantage can delay evolution to “optimal virulence” [28,56] that maximizes transmissibility, and can also delay the spread of drug resistance (by onward transmission [57]) in the face of increasing selection pressure from the broadening scope of ART. Widely available ART may affect resident and invader strains equally, effectively reducing the baseline rate of transmissions and transforming a high-prevalence setting towards lower prevalence. Given that most aspects of the first comer advantage were strong in both low- and high-prevalence settings in our simulations, the broadening scope of ART may not affect this phenomenon strongly.

Because the mechanisms of first comer advantage do not operate at the front wave of an epidemic expanding into a susceptible population, we suggest that much of the (non-local) adaptation of HIV may have happened along these front waves, rather than in populations where prevalence has stabilized. Furthermore, considering that the currently dominant subtypes probably all expanded riding the wave of their first comer advantage, most or all of them may in fact possess suboptimal fitness and transmissibility. If the original founder strains of the early expansions were selected (at least partly) by “chance”, rather than due to high fitness, then even subsequent evolution may have constrained the subtypes to the local suboptima of the fitness landscape that were accessible from the initial sequence. This implies that the initial founder effects and the first comer advantage may have provided some benefit by preventing the fast global spread of the most transmissible HIV variants in the growth phase of the pandemic. However, the results also caution that the next stage of the pandemic may be characterized by a shift towards more transmissible strains over the slow time scales predicted by our model, and data from several regions indicate that this process has already started. HIV-1 subtype A is spreading at the expense of subtype D in Eastern Africa [5,25,26], and HIV-1 is expanding at the expense of HIV-2 in Western Africa [27,58]. Our results suggest that these relatively fast replacements require a large selective advantage of the expanding strain. Indeed, subtype A is associated with higher transmissibility [12] and slower disease progression [14,15] compared with subtype D, and HIV-2 has two orders of magnitude lower replicative capacity [59] and more than 3-fold lower per contact transmissibility [60] compared with HIV-1. In comparison, within individual patients the replicative fitness (a probable correlate of transmissibility) showed only about 10% variation between the fittest and the average viral genome in a study of untreated HIV-1 infected patients [61]. Our results indicate that variations of greater magnitude are needed to drive the relatively fast replacement dynamics of the few observed cases.

While differences are expected between the currently characterized subtypes, and those more efficient at transmission are slowly gaining ground at the expense of less transmissible subtypes, major innovations and potentially higher transmissibility may be more likely to arise from the complex diversity of HIV in Central Africa and from the recombinant forms. CRFs probably emerged against the backdrop of established epidemics and their growth to detectable levels may indicate considerable selection advantage. The rapid growth of several CRFs in recent years [5,62] is consistent with this concept and is therefore cause for concern for the future of the pandemic.

The interference mechanisms and first comer advantage demonstrated in this paper may also help explain why so few cross-species transmissions of SIV to humans were able to establish epidemic HIV lineages, and why no new major HIV types or groups have emerged since the middle of the 20^th^ century [63]. It is possible that a successful reduction or elimination of the current HIV epidemic in Africa may, by eliminating the inhibiting competition effects, increase the risk for the emergence of new HIV lineages from novel cross-species transmissions.

Finally, we note that HIV may represent a rare combination of factors relevant for the observed first comer advantage: infection lasts and remains active for life; the inhibition of superinfection does not seem to be (strongly) strain specific [64] (as opposed to other infections with serotypes that elicit type specific immunity); and infected individuals remain in the contact network for many years. Taken together, these factors may imply that the first comer advantage, and its consequence of delayed global mixing, may be particularly strong for HIV and weaker for most other pathogens. For example, a persistent infection controlled by strain specific immunity would correspond approximately to our HIV scenario with no inhibition of superinfection, in which case most of the population level effect was lost in the simulations. Non-persistent infections would tip the balance further in favour of the novel strain, because individuals recovered from the initial infection would be susceptible to the novel strain while ceasing to transmit or be susceptible to the first strain.

In all, our results suggest that the interference mechanisms of competition, possibly aided by local adaptation, can slow down the adaptation of HIV at the population level, in spite of the huge evolutionary potential of the virus. These effects may explain why strong founder effects still persist several decades after the initial global expansion of the pandemic, and may hamper the ongoing adaptation of the virus to maximize its transmissibility and also slow down the spread of drug resistance.

## Materials and Methods

### Simulation model

We developed a stochastic, individual-based simulation model to track the spread of HIV over a dynamic network of heterosexual contacts. The network consisted of three types of nodes (individuals): males, females and female sex workers (FSW). The model tracked the age and HIV status (stage of infection and the infecting virus type) of each individual, and for males and (non-FSW) females also a fixed quantifier of promiscuity (preferred annual contact degree), and the number of distinct sexual partners in the last year (realized annual contact degree). Individuals entered the population at age 15 and were removed at age 50. The preferred contact degree of each individual was drawn from an empirical distribution according to the type of the node and was kept constant for the lifetime of the individual. The promiscuity of males and (non-FSW) females was characterized by continuous power-law distributions of the form P(x) ∼ x^-γ^ (with different exponents for the two sexes) parameterized based on empirical data (Table 1), and censored at both a lower cut-off (one contact per year to ensure all nodes are active in the network) and an upper cut-off. FSW had a fixed maximum number of one-time contacts per week.

The simulations had a time step of one week, and each step consisted of the following procedures: a) generation of sexual acts along the links and virus transmission, b) update of HIV status, c) birth and death dynamics of individuals, d) dissolution and formation of network links. The number of sexual acts in male-female links was drawn from a Poisson distribution (discarding zeros: no links were inactive); male-FSW links always involved a single sexual act. The probability of virus transmission to uninfected individuals was determined by the baseline transmission rate of the virus strain, amplified if the transmitting individual was in the acute stage of the infection. Newly infected individuals were immediately assigned a time to death from a uniform distribution between 3–20 years (consistent with recently estimated survival curves in ART-naïve cohorts [65]), and for each infection event we recorded: the date of the event, the strain that was transmitted, the disease stage of the transmitter, and whether the transmission involved superinfection of an individual previously infected with the other virus type. For simplicity, the size of the population was kept constant (at 10,000 individuals of both sexes): all nodes who died of AIDS or left the network at age 50 (whichever came first) were replaced with a new individual of age 15. The preferred annual contact degree of new nodes was drawn from the power-law distribution of the respective gender at entry to the population. The links between males and females were allowed to form and break up at each time step. The baseline probability of break-up was set to yield an average duration consistent with empirical estimates (Table 1), and was scaled proportional to the average contact degree of the two nodes (such that more promiscuous individuals had shorter relationships [66]). Link formation was implemented such that all non-FSW individuals would have a yearly number of sexual contacts approximately equivalent to their preferred annual contact degree. At each time step, the nodes were assigned a number of half-links generated randomly in proportion to their preferred contact degree. Because males had greater mean promiscuity than non-FSW females, the number of half-links for males exceeded those of the females. New links were formed by first randomly connecting all female half-links to male half-links, then randomly distributing the remaining male half-links to the FSW. All runs were started with an initialization phase restricted to link formation and break-up until the sexual network settled to a steady state. FSWs had fixed promiscuity and were added one by one as long as there was a surplus of male half-links. The number of FSWs at steady-state was thus not pre-determined, but emerged to match and compensate the imbalance of male and (non-FSW) female links in each scenario.

Population level competition was simulated by implementing two virus types that were allowed to differ in their rate of transmissibility. The type of the infecting virus strain(s) was tracked for each infected individual. The first virus strain was introduced in a random sample of ten percent of all FSW after the initialization of the sexual network: this method allowed a reliable establishment of the “resident” epidemic with negligible risk of extinction. The second (invader) strain was also introduced in a sample of ten percent of all FSW (sampled from uninfected FSW) when the resident strain has attained a steady state in the population.

In the default scenario, superinfection could occur only by the replacement of the original strain with the superinfecting strain. In a sexual act between two individuals infected with different virus strains, both strains had a chance to be transmitted. Superinfection occurred if two check points were passed: initial transmission occurred according to the transmission rate of the infecting strain (modified by disease stage, if appropriate); then after successful initial transmission, the probability of superinfection was determined by the relative transmission rates (“fitness”) of the two strains as follows: P = (ν_2_/(ν_1_+ν_2_)), where ν_1_ denotes the transmission rate of the virus infecting the potential recipient and ν_2_ denotes the transmission rate of the strain infecting the potential transmitter. The “clock” of disease stage in the recipient was unaffected by superinfection in the default scenario; the stage of disease remained to be based on the age of infection defined by the date of the original first infection of the recipient. A new time to death was drawn randomly (from the 3–20 years range); however, it was used only if the new date of death preceded the original date determined at the initial infection: superinfection could never extend the lifespan of an individual.

We implemented three scenarios to test the potential mechanisms of interference. In the “dual infection” scenario, superinfection occurred at the same rate as initial infection (i.e., according to the transmission rate of the superinfecting strain), and the two strains co-existed in the superinfected individuals. Superinfected (dually infected) individuals were then able to transmit both virus strains independently in subsequent contacts. We did not implement a scenario in which superinfection was unhindered but co-existence not allowed, because such a situation would imply an asymmetric advantage for the superinfecting strain. In the “multiple acute” scenario, the clock of disease stage was reset upon superinfection, and the superinfecting strain started a new episode of peak acute-stage transmissibility (however, this new episode could not extend the lifespan of the individual beyond his/her original date of death, determined after the first infection). Finally, in the “fixed degrees” scenario, new individuals were added with a preferred contact degree identical to that of the individual whom they replaced after his/her death due to AIDS.

The parameters of the sexual network were based on contemporary surveys in Africa; HIV parameters were also based on available empirical data (Table 1). The high-prevalence setting was implemented by increasing (doubling) the baseline transmission rate, consistent with the recent finding that variation in prevalence among Sub-Saharan countries can largely be explained by differences in the rate of transmission in serodiscordant couples [67].

The model was implemented in the C++ programming language. The full computer code of the simulations is available in S1 File.

### Statistics

Statistical tests were performed with R [68]. Power-law exponents of the realized annual contact degrees (based on the actual numbers of sexual contacts in the last year) were fitted as described in [69], estimating the lower cutoff with Kolmogorov-Smirnov statistics, using the implementation of [70].

## Supporting Information

S1 File Computer code of simulations. (ZIP)

S1 Fig The growth of the invader strain at different values of relative advantage in the transmission rate. The relative advantage of the invader virus was varied from zero (top row) to 50% (bottom row) in the low (left column) or high (right column) prevalence scenarios. The resident strain (solid purple line) was introduced in the population at Week 1000 (to allow the network to attain steady state); the invader strain (dashed green line) was introduced in the population when the first had already reached steady-state prevalence (at Week 5000 and 7000 for the high- and low-prevalence setting, respectively). The lines show median prevalence from simulations where the invader strain did not go extinct (out of 1000 simulation runs); shading indicates the areas between the 5% and 95% quantiles. Simulation parameters were set as in Table 1; superinfection and replacement dynamics followed the default scenario.
(PDF)

S2 Fig The relative contribution of acute stage transmissions over the time course of single-strain epidemics. The proportion of transmissions originating from acute-stage transmitters decreases from high levels at the beginning of the epidemics to a steady-state around 0.15 and 0.13 in the low (purple dots) and high (green dots) prevalence epidemics, respectively, over a time scale of a few decades. Proportion data were calculated by combining transmission events recorded in 1000 simulation runs, then smoothed by averaging with a sliding window of length 100 weeks. Parameters were set as in Table 1; superinfection and replacement dynamics followed the default scenario.
(PDF)

S3 Fig The probability of infection as a function of the promiscuity (preferred contact degree) of the individuals: data and model fit. Using collated data from 100 simulation runs (2 million individuals total), we performed a logistic regression on the probability of infection in individuals using log transformed preferred contact degree, age and gender as explanatory variables. Purple and red lines show smoothed actual proportions of infecteds among females and males, respectively, calculated with a sliding window (moving along all individuals sorted according to contact degree; each point representing the frequency of infections among 1000 individuals). Predictions from the logistic regression (plotted as orange and green lines, using the same sliding window smoothing) provide an excellent fit to the data. Effect sizes (and 95% CI) for the three factors were estimated as follows: log10(degree): 2.48 (95% CI: 2.46–2.50), age: 0.0460 per year (95% CI: 0.0457–0.0464), female gender: 0.420 (95% CI: 0.413–0.428); all three effects were significant at p<10^-10^. Parameters were set as in Table 1; superinfection and replacement dynamics followed the default scenario.
(PDF)

S4 Fig Preferential depletion of highly promiscuous individuals among non-FSW females. (A) The frequency distribution of the annual number of sexual contacts (realized contact degree) in females in uninfected populations (purple dots) and in populations with high-prevalence epidemics (green squares), based on median data from 1000 simulation runs. Highly promiscuous individuals were selectively depleted in the presence of the virus. (B) Boxplot of the exponents of power-law distributions fitted to female individuals in batches of 1000 independent runs with no virus, low and high prevalence epidemics, respectively. Boxes depict interquartile range, median is indicated by horizontal lines within the boxes, and whiskers extend to the farthest values that are not more than 1.5 times the box width away from the box. Medians (and IQR) of the exponents were 3.86 (3.73–3.97), 3.99 (3.83–4.09) and 4.19 (3.99–4.32) in the absence of the virus and with low or high prevalence epidemics, respectively; all pairwise comparisons between the three scenarios were statistically significant (p<10^–10^; Wilcoxon rank sum test). Simulation parameters were set as in Table 1; superinfection and replacement dynamics followed the default scenario.
(PDF)

S5 Fig The effect of multiple acute infections on the competition of HIV strains. The figure compares the outgrowth of an invader virus with 5% transmission rate advantage in the high (top row) and low (bottom row) prevalence settings with default superinfection dynamics (left: A, C) or with repeated peaks of acute-stage infectiousness upon superinfection (right: B, D). The resident strain (solid purple line) was introduced in the population at Week 1000 (to allow the network to attain steady state); the invader strain (dashed green line) was introduced in the population when the first strain had already attained steady-state prevalence (at Week 5000 and 7000 for the high- and low-prevalence setting, respectively). Multiple acute peaks accelerate the outgrowth of the invader strain and the decline of the resident considerably in the high prevalence scenario (A vs. B), but not in the low prevalence scenario (C vs. D), where superinfection is rare. The lines show median prevalence from simulations where the invader strain did not go extinct (out of 1000 simulation runs); shading indicates the areas between the 5% and 95% quantiles. Simulation parameters were set as in Table 1; scenarios are described in detail in the main text.
(PDF)

S6 Fig The effect of population turnover on the “first comer advantage”. All quantifiers are plotted against the relative transmission rate advantage of the second (invader) strain, for two levels of population turnover: 35 (default, purple dots) or 20 years (red squares) of uninfected (sexually active) lifespan, in the high (top row) and low (bottom row) prevalence settings. Faster turnover had little effect on the probability of extinction of the invader strain, but could have a pronounced effect on its rate of growth at low values of the transmission advantage. Data in B-C and E-F depict medians from 1000 simulation runs (excluding those where the invader virus went extinct). Parameters are listed in Table 1; superinfection and replacement dynamics followed the default scenario. The maximum length of simulations was 19,000 weeks (~365 years); empty symbols indicate where the invader strain did not reach the threshold prevalence by the end of the simulation in the majority of the cases.
(PDF)
