# Supplementary figures and images for: HIV Competition Dynamics over Sexual Networks: First Comer Advantage Conserves Founder Effects
Source: PLoS Comput Biol. 2015 Feb 5;11(2):e1004093. doi: 10.1371/journal.pcbi.1004093 (PMC4318579; doi:10.1371/journal.pcbi.1004093)

— first strain  
low prevalence

- - second strain  
high prevalence

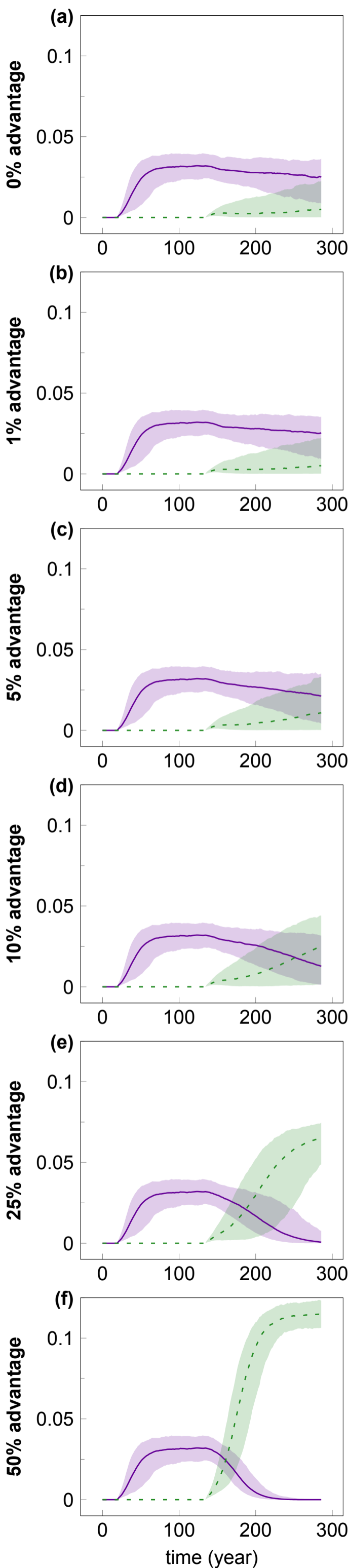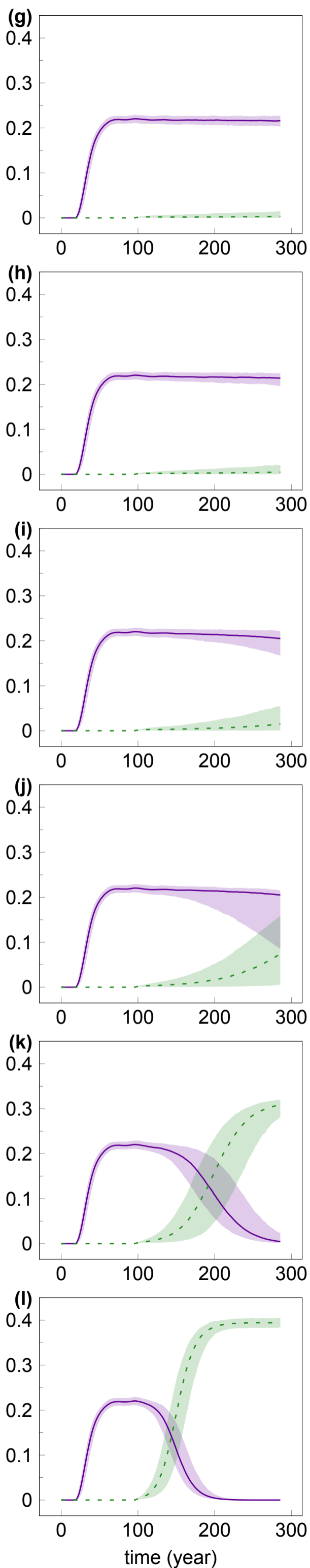

Supplement: S1 Fig — The relative advantage of the invader virus was varied from zero (top row) to 50% (bottom row) in the low (left column) or high (right column) prevalence scenarios. The resident strain (solid purple line) was introduced in the population at Week 1000 (to allow the network to attain steady state); the invader strain (dashed green line) was introduced in the population when the first had already reached steady-state prevalence (at Week 5000 and 7000 for the high- and low-prevalence setting, respectively). The lines show median prevalence from simulations where the invader strain did not go extinct (out of 1000 simulation runs); shading indicates the areas between the 5% and 95% quantiles. Simulation parameters were set as in Table 1; superinfection and replacement dynamics followed the default scenario. (PDF) [file pcbi.1004093.s002.pdf]

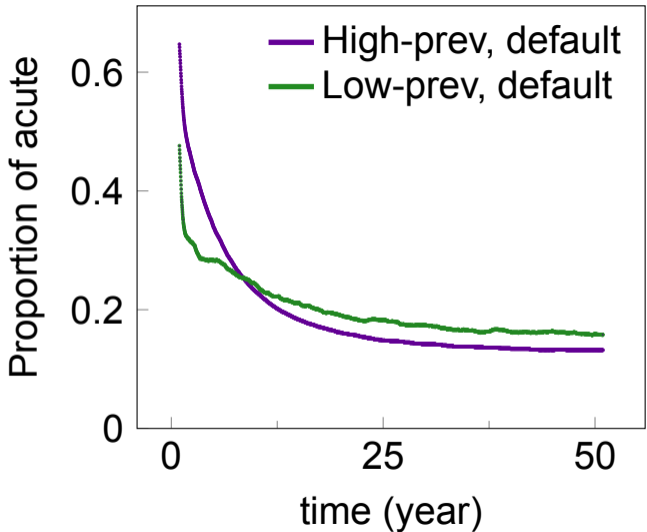

Supplement: S2 Fig — The proportion of transmissions originating from acute-stage transmitters decreases from high levels at the beginning of the epidemics to a steady-state around 0.15 and 0.13 in the low (purple dots) and high (green dots) prevalence epidemics, respectively, over a time scale of a few decades. Proportion data were calculated by combining transmission events recorded in 1000 simulation runs, then smoothed by averaging with a sliding window of length 100 weeks. Parameters were set as in Table 1; superinfection and replacement dynamics followed the default scenario. (PDF) [file pcbi.1004093.s003.pdf]

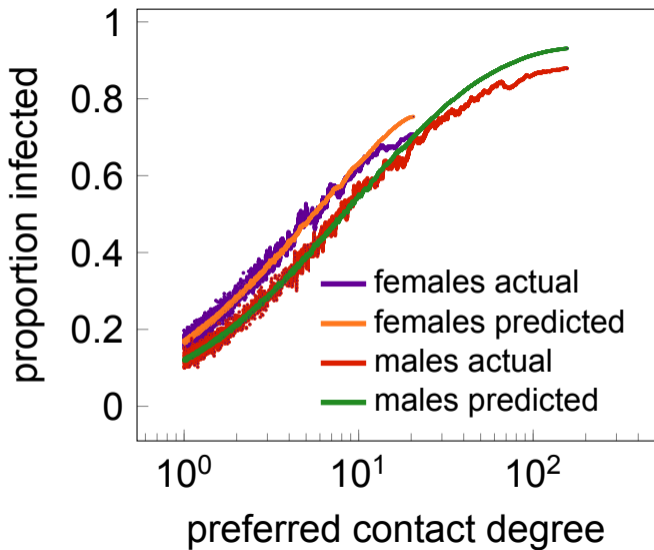

Supplement: S3 Fig — Using collated data from 100 simulation runs (2 million individuals total), we performed a logistic regression on the probability of infection in individuals using log transformed preferred contact degree, age and gender as explanatory variables. Purple and red lines show smoothed actual proportions of infecteds among females and males, respectively, calculated with a sliding window (moving along all individuals sorted according to contact degree; each point representing the frequency of infections among 1000 individuals). Predictions from the logistic regression (plotted as orange and green lines, using the same sliding window smoothing) provide an excellent fit to the data. Effect sizes (and 95% CI) for the three factors were estimated as follows: log10(degree): 2.48 (95% CI: 2.46–2.50), age: 0.0460 per year (95% CI: 0.0457–0.0464), female gender: 0.420 (95% CI: 0.413–0.428); all three effects were significant at p<10-10. Parameters were set as in Table 1; superinfection and replacement dynamics followed the default scenario. (PDF) [file pcbi.1004093.s004.pdf]

frequency distribution

(a)

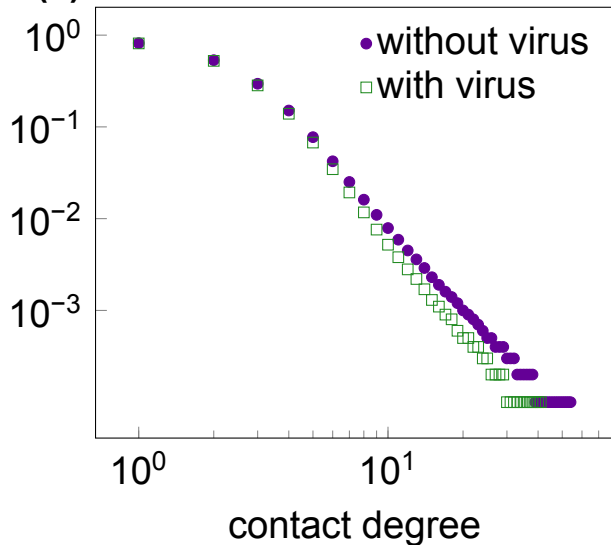

fitted exponent

(b)

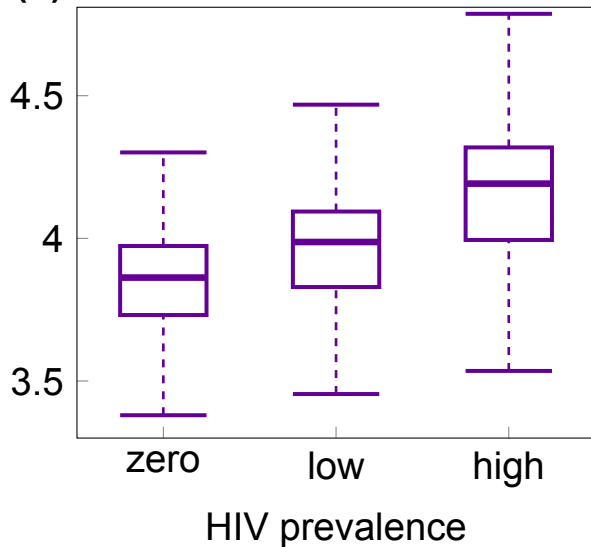

Supplement: S4 Fig — (A) The frequency distribution of the annual number of sexual contacts (realized contact degree) in females in uninfected populations (purple dots) and in populations with high-prevalence epidemics (green squares), based on median data from 1000 simulation runs. Highly promiscuous individuals were selectively depleted in the presence of the virus. (B) Boxplot of the exponents of power-law distributions fitted to female individuals in batches of 1000 independent runs with no virus, low and high prevalence epidemics, respectively. Boxes depict interquartile range, median is indicated by horizontal lines within the boxes, and whiskers extend to the farthest values that are not more than 1.5 times the box width away from the box. Medians (and IQR) of the exponents were 3.86 (3.73–3.97), 3.99 (3.83–4.09) and 4.19 (3.99–4.32) in the absence of the virus and with low or high prevalence epidemics, respectively; all pairwise comparisons between the three scenarios were statistically significant (p<10–10; Wilcoxon rank sum test). Simulation parameters were set as in Table 1; superinfection and replacement dynamics followed the default scenario. (PDF) [file pcbi.1004093.s005.pdf]

— first strain  
default w/ 5% advantage

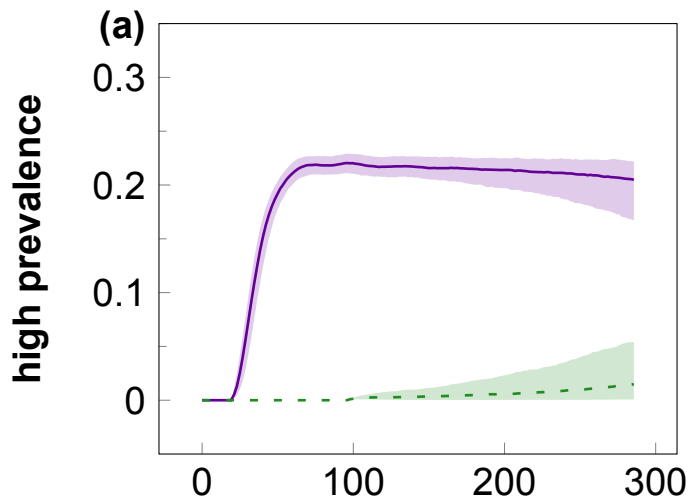

- - second strain  
multiple acute w/ 5% advantage

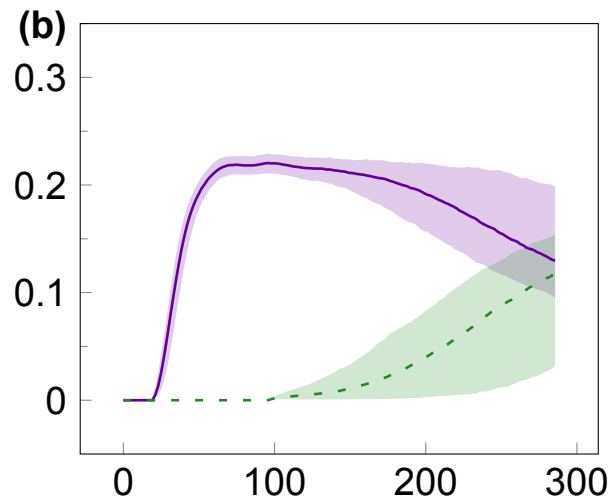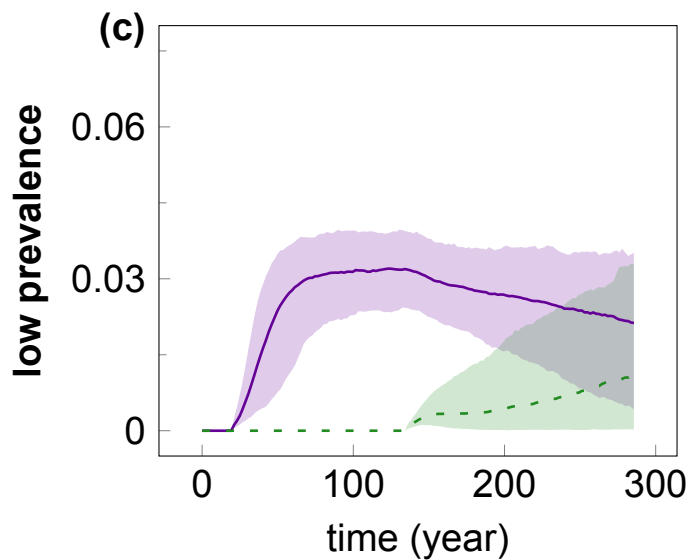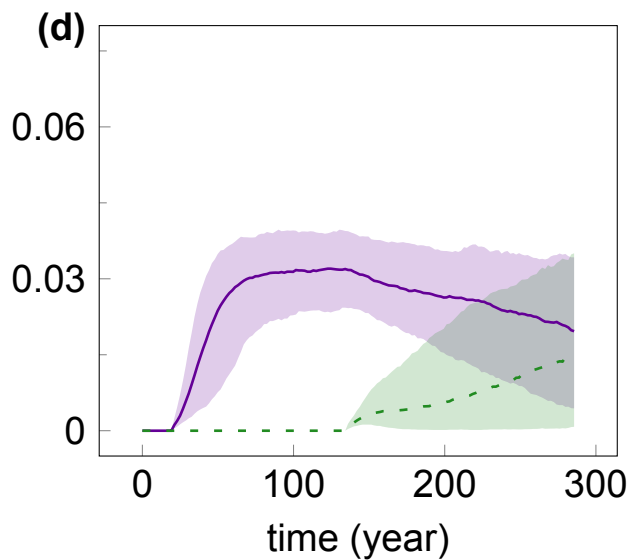

Supplement: S5 Fig — The figure compares the outgrowth of an invader virus with 5% transmission rate advantage in the high (top row) and low (bottom row) prevalence settings with default superinfection dynamics (left: A, C) or with repeated peaks of acute-stage infectiousness upon superinfection (right: B, D). The resident strain (solid purple line) was introduced in the population at Week 1000 (to allow the network to attain steady state); the invader strain (dashed green line) was introduced in the population when the first strain had already attained steady-state prevalence (at Week 5000 and 7000 for the high- and low-prevalence setting, respectively). Multiple acute peaks accelerate the outgrowth of the invader strain and the decline of the resident considerably in the high prevalence scenario (A vs. B), but not in the low prevalence scenario (C vs. D), where superinfection is rare. The lines show median prevalence from simulations where the invader strain did not go extinct (out of 1000 simulation runs); shading indicates the areas between the 5% and 95% quantiles. Simulation parameters were set as in Table 1; scenarios are described in detail in the main text. (PDF) [file pcbi.1004093.s006.pdf]
