# Supplementary material for: HIV Competition Dynamics over Sexual Networks: First Comer Advantage Conserves Founder Effects
Source: PLoS Comput Biol. 2015 Feb 5;11(2):e1004093. doi: 10.1371/journal.pcbi.1004093 (PMC4318579; doi:10.1371/journal.pcbi.1004093)
Supplement: S6 Fig — All quantifiers are plotted against the relative transmission rate advantage of the second (invader) strain, for two levels of population turnover: 35 (default, purple dots) or 20 years (red squares) of uninfected (sexually active) lifespan, in the high (top row) and low (bottom row) prevalence settings. Faster turnover had little effect on the probability of extinction of the invader strain, but could have a pronounced effect on its rate of growth at low values of the transmission advantage. Data in B-C and E-F depict medians from 1000 simulation runs (excluding those where the invader virus went extinct). Parameters are listed in Table 1; superinfection and replacement dynamics followed the default scenario. The maximum length of simulations was 19,000 weeks (~365 years); empty symbols indicate where the invader strain did not reach the threshold prevalence by the end of the simulation in the majority of the cases. (PDF) [file pcbi.1004093.s007.pdf]

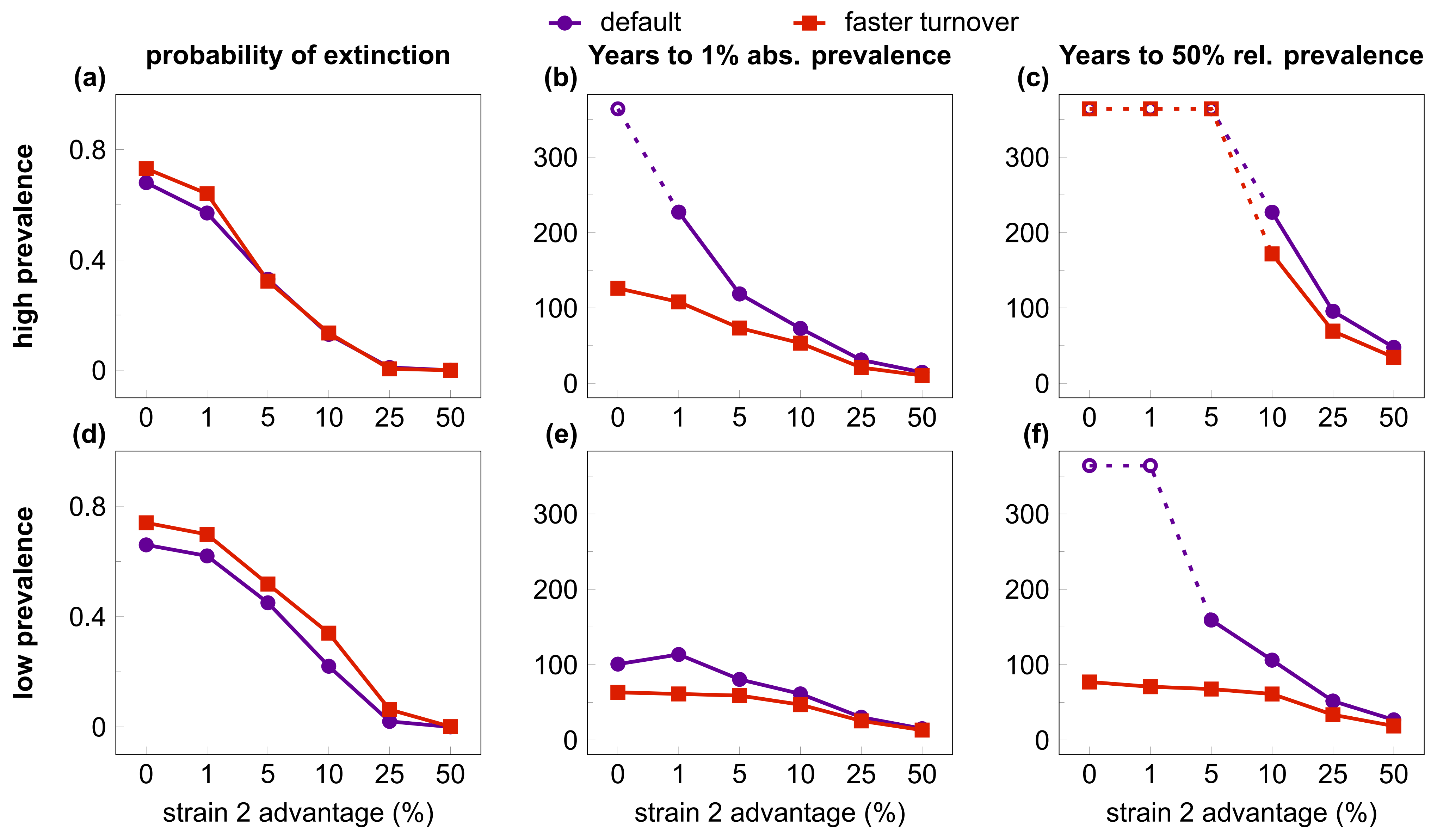

default
faster turnover
probability of extinction
Years to 1% abs. prevalence
Years to 50% rel. prevalence
(a)
(b)
(c)
(d)
(e)
(f)
high prevalence
low prevalence
0.8
0.4
0
300
200
100
0
0
1
5
10
25
50
strain 2 advantage (%)
